# Synthese von α‐Aminosäurederivaten durch hydrative Aminierung von Alkinen

**DOI:** 10.1002/ange.202212399

**Published:** 2022-11-30

**Authors:** Minghao Feng, Roberto Tinelli, Ricardo Meyrelles, Leticia González, Boris Maryasin, Nuno Maulide

**Affiliations:** ^1^ Institut für Organische Chemie Universität Wien Währinger Straße 38 1090 Wien Österreich; ^2^ Institut für Theoretische Chemie Universität Wien Währinger Straße 17 1090 Wien Österreich; ^3^ Vienna Doctoral School in Chemistry Universität Wien Währinger Straße 42 1090 Wien Österreich

**Keywords:** Aminierung, Aminosäuren, Inamide, Reaktionsmechanismen, Umlagerungen

## Abstract

α‐Aminosäurederivate sind Kernbestandteile jeglichen Lebens. Die Synthese von α‐Aminocarbonyl/carboxyl Verbindungen ist jedoch weiterhin eine Herausforderung für die organische Synthese. In dieser Arbeit berichten wir von einer praktischen Herstellungsmethode für α‐Aminosäurederivate durch direkte hydrative Aminierung von aktivierten Alkinen mit Sulfinamiden unter milden Bedingungen. Computergestützte Untersuchungen legen nahe, dass eine [2,3]‐sigmatrope Sulfoniumumlagerung der zentrale Schritt der Reaktion ist.

## Graphical abstract

α‐Aminosäurederivate sind Grundbausteine jeglichen Lebens. Hierin berichten wir von einer hydrativen Aminierung von Inamiden und Thioalkinen unter metallfreien und milden Bedingungen, welche diese Bausteine unter Verwendung von kommerziell erhältlichen Sulfinamiden auf einfache Art und Weise herstellt. Computergestützte Berechnungen legen nahe, dass die Reaktion auf einer neuen Art der [2,3]‐sigmatropen Sulfoniumumlagerung beruht.

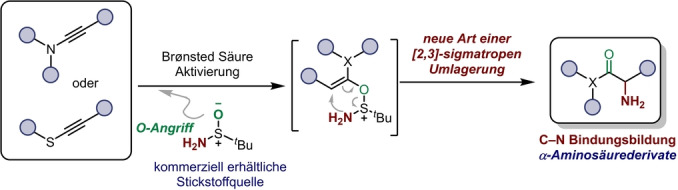

α‐Aminosäurederivate sind Grundbausteine der Natur und kommen in vielen pharmazeutisch wirksamen Verbindungen und Naturstoffen vor.^[1]^ Aufgrund ihrer Rolle in der Modulierung von Peptideigenschaften ist die Suche nach neuen Methoden zur Herstellung nicht‐natürlicher α‐Aminocarbonylderivative weiterhin von großer Bedeutung.[^2^, ^3^] Die hydrative Funktionalisierung von Alkinen hat sich in den letzten Jahren als eine effiziente Strategie zur Herstellung α‐funktionalisierter Carbonylverbindungen herauskristallisiert.^[4]^ Viele verschiedene Methoden basierend auf Brønstedsäurekatalyse[^5^, ^6^] oder Übergangsmetallkatalyse^[7]^ wurden entwickelt und führen zu unterschiedlichsten α‐substituierten Carbonylen. Die Protokolle zur Synthese von α‐Aminocarbonylen, jedoch, leiden oft unter Einschränkungen in Bezug auf die Stickstoffquelle,^[8]^ oder bedürfen mehrerer Stufen.[^7a^, ^9^]

Unsere Gruppe, wie auch andere, hat Methoden für die hydrative Funktionalisierung von Alkinen (zum Beispiel hydrative Arylierung und Alkylierung) entwickelt, die auf [3,3]‐sigmatropen Umlagerungen basieren (Schema 1A).[^10^, ^11^] Im Gegensatz dazu sind [2,3]‐sigmatrope Umlagerungen in diesem Kontext weniger geläufig,^[12]^ trotz der Publikation einer bahnbrechenden Aminierungsmethode durch Sharpless im Jahr 1976 (Schema 1B).^[13]^ In dieser Arbeit wurden Allylamine durch eine Sequenz von En‐Reaktion/[2,3]‐sigmatroper Umlagerung hergestellt.^[14]^ In der Hoffnung eine generelle Methode für die Herstellung von α‐Aminocarbonylen durch hydrative Aminierung zu entwickeln, machten wir uns die Eigenschaften geläufiger Sulfinamide (zum Beispiel das kommerziell erhältliche *t*‐Butylsulfinamid **2**) in Kombination mit aktivierten Alkinen zu Nutze. Wie in Schema 1C gezeigt, spekulierten wir, dass das entscheidende Intermediat **Int I** eine [2,3]‐sigmatrope Umlagerung eingehen könnte, wodurch die gewünschten α‐Aminocarbonylderivate gebildet würden. Obwohl die Kompatibilität des Sulfinamids mit den Brønsted‐sauren Bedingungen, sowie die Aufarbeitung der mutmaßlichen N−S Bindung des Zwischenprodukts fraglich waren, wurden wir von der Möglichkeit einer direkten Synthese von α‐Aminocarbonylverbindungen angetrieben, unsere Untersuchungen zu starten. Vielleicht das größte Fragezeichen stellte die Überlegung dar, ob der *Stickstoff* oder der *Sauerstoff* des Sulfinamids das elektrophile Intermediat angreifen würden. Diese Frage ließ sich am besten durch das Experiment beantworten.

**Scheme 1:**
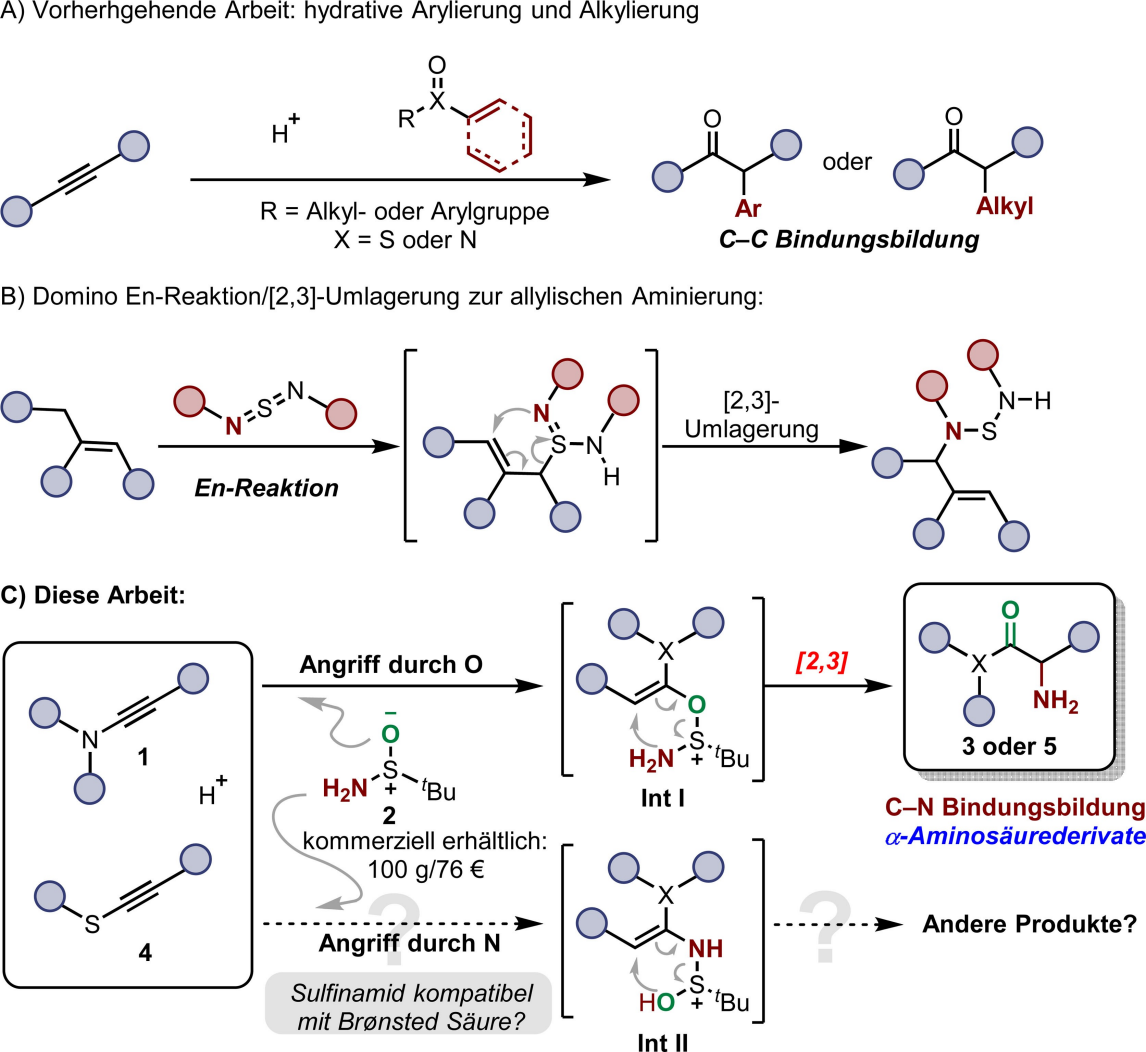
A) Vorhergehende Arbeit über hydrative Arylierung und Alkylierung von Alkinen mittels [3,3]‐sigmatroper Umlagerung. B) [2,3]‐Sigmatrope Umlagerung zur allylischen Aminierung. C) Diese Arbeit: Synthese von α‐Aminosäurederivaten durch hydrative Aminierung mittels [2,3]‐sigmatroper Sulfoniumumlagerung und mögliche Konkurrenz durch Angriff des Stickstoffs.

Anfangs brachten wir 3‐(Hept‐1‐in‐1‐yl)oxazolidin‐2‐on **1 a** mit kommerziell erhältlichem *t*‐Butylsulfinamid **2** in der Gegenwart einer Brønsted Säure zur Reaktion. Wie in Tabelle 1 ersichtlich, sind Trifluoressigsäure und *p*‐Toluolsulfonsäure nicht in der Lage die Inamide auf gewünschte Art und Weise zu aktivieren (Einträge 1 & 2). Das gewünschte Produkt **3 a** konnte jedoch, nach in situ Benzoyl‐Schützung, in guter Ausbeute isoliert werden, als Trifluormethansulfonsäure (Eintrag 3) oder Bis(trifluormethan)sulfonimid (Eintrag 4) verwendet wurden. Nur Spuren von **3 a** wurden detektiert als die Reaktionstemperatur gesenkt wurde (Eintrag 5) oder lediglich katalytische Mengen der Säure eingesetzt wurden (Eintrag 6). Weiters wurde die Präaktivierung des Inamids als entscheidender Faktor ausgemacht, da dadurch eine konkurrierende Reaktion durch das basische Sulfonamid ausgeschlossen werden kann (Eintrag 7).

**Table 1:**
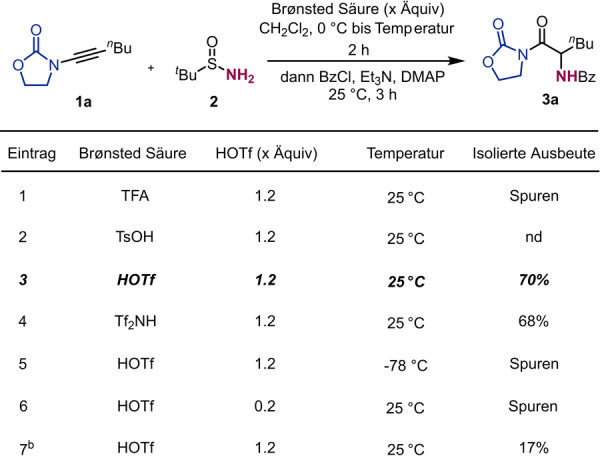
Optimierung der hydrativen Aminierung von Inamid **1 a**.^[a]^

[a] **1 a** (0.2 mmol, 1.0 Äquiv. in 1.0 mL CH_2_Cl_2_), Brønsted Säure (1.2 Äquiv.) bei 0 °C für 15 min, dann **2** (2.0 Äquiv. in 1.0 mL CH_2_Cl_2_). Nach 2 h, Et_3_N (3.0 Äquiv.), DMAP (5 mol %) und BzCl (3.0 Äquiv.), 25 °C für 3 h. [b] Das Sulfinamid wurde direkt nach Zugabe von HOTf zugegeben. TFA: Trifluoressigsäure. HOTf: Trifluormethansulfonsäure. Tf_2_NH: Bis(trifluormethan)sulfonimid.

Mit den optimalen Reaktionsbedingungen starteten wir unsere Untersuchungen der Substratbreite der Reaktion. Wie in Schema 2 gezeigt, lieferte eine Reihe von Inamiden mit diversen Alkylsubstituenten die gewünschten Produkte (**3 a**–**3 d**) in guten Ausbeuten. Die Struktur des Produkts **3 a** konnte durch Röntgenkristallographie bestätigt werden (CCDC 2171644).^[21]^ Es ist hervorzuheben, dass die Einführung von Halogeniden, Phthalimiden und konjugierten Alkenen die Effizienz des Prozesses nicht negativ beeinflusste (**3 e**, **3 f** und **3 i**). Ebenso kann die Reaktion an Substraten mit ausgeprägter sterischer Hinderung (**3 g**) durchgeführt werden und ermöglicht sogar die Aminieurng neopentylischer Positionen (**3 h**). Inamide mit aromatischen Substituenten lieferten die gewünschten Produkte (**3 j**–**3 t**) ebenso in guten bis ausgezeichneten Ausbeuten. Des Weiteren ist ein Inamidderivat von Febuxostat hervorzuheben, welches das entsprechende aminierte Produkt (**3 u**) in guter Ausbeute bildete.

**Scheme 2:**
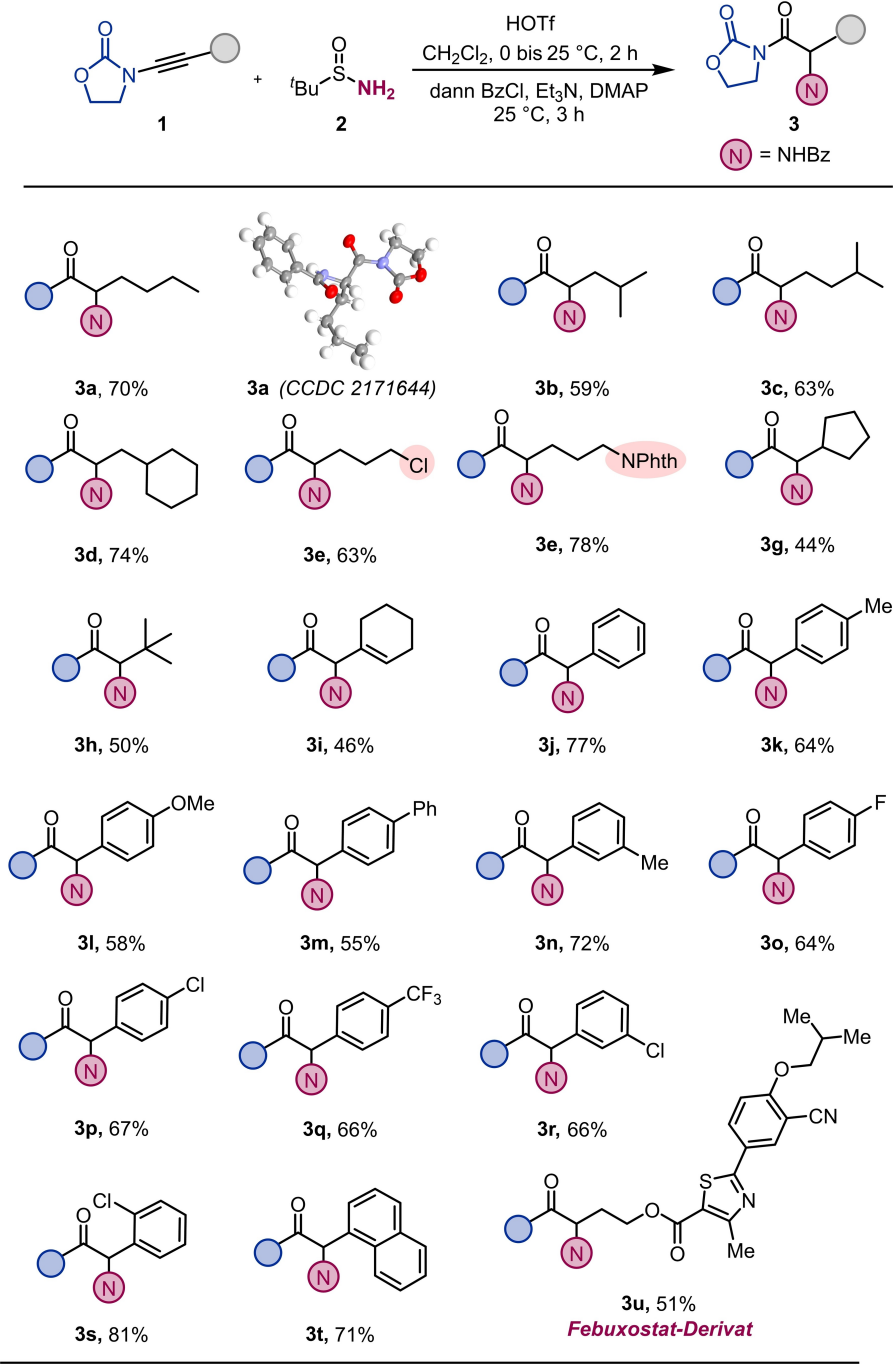
Hydrative Aminierung von Inamiden: **1** (0.2 mmol, 1.0 Äquiv. in 1.0 mL CH_2_Cl_2_), HOTf (1.2 Äquiv.) bei 0 °C für 15 min, dann **2** (2.0 Äquiv. in 1.0 mL CH_2_Cl_2_). Nach 2 h, Et_3_N (3.0 Äquiv.), DMAP (5 mol %) und BzCl (3.0 Äquiv.), 25 °C für 3 h.

Durch die Resultate mit Inamiden ermutigt, wandten wir uns den verwandten Thioalkinen zu. Der Übergang zu dieser Substratklasse benötigte einiges an Optimierung der Reaktionsbedingungen (siehe Hintergrundinformation). Die resultierenden Reaktionsbedingungen, unter anderem längere Reaktionszeiten und geringere Mengen Trifluormethansulfonsäure, ermöglichten die Bildung des Modellprodukts **5 a** in reproduzierbaren und hohen Ausbeuten (Schema 3). Im Zuge weiterer Untersuchungen zeigte sich, dass einige der Substrate von der Verwendung von Bis(trifluormethan)sulfonimid profitierten und die entsprechenden α‐Aminothioester in höhreren Ausbeuten erhalten werden konnten. Thioalkine mit einer Reihe von (zyklo)aliphatischen Resten konnten so effizient in die gewünschten Produkte überführt werden (**5 a**–**5 g**). Wie schon bei Inamiden, toleriert auch diese Reaktion viele funktionelle Gruppen, wie zum Beispiel Halogenide (**5 h**), Acetate (**5 i**) und stickstoffhältige Gruppen (**5 j**). Substrate mit ausgeprägter sterischer Hinderung (**4 k**), wie auch solche mit aromatischen Substituenten (**4 l**, **4 m**) und ein *S*‐Arylthioalkin (**4 n**) lieferten die gewünschten Produkte (**5 k**–**5 n**) ebenso.

**Scheme 3:**
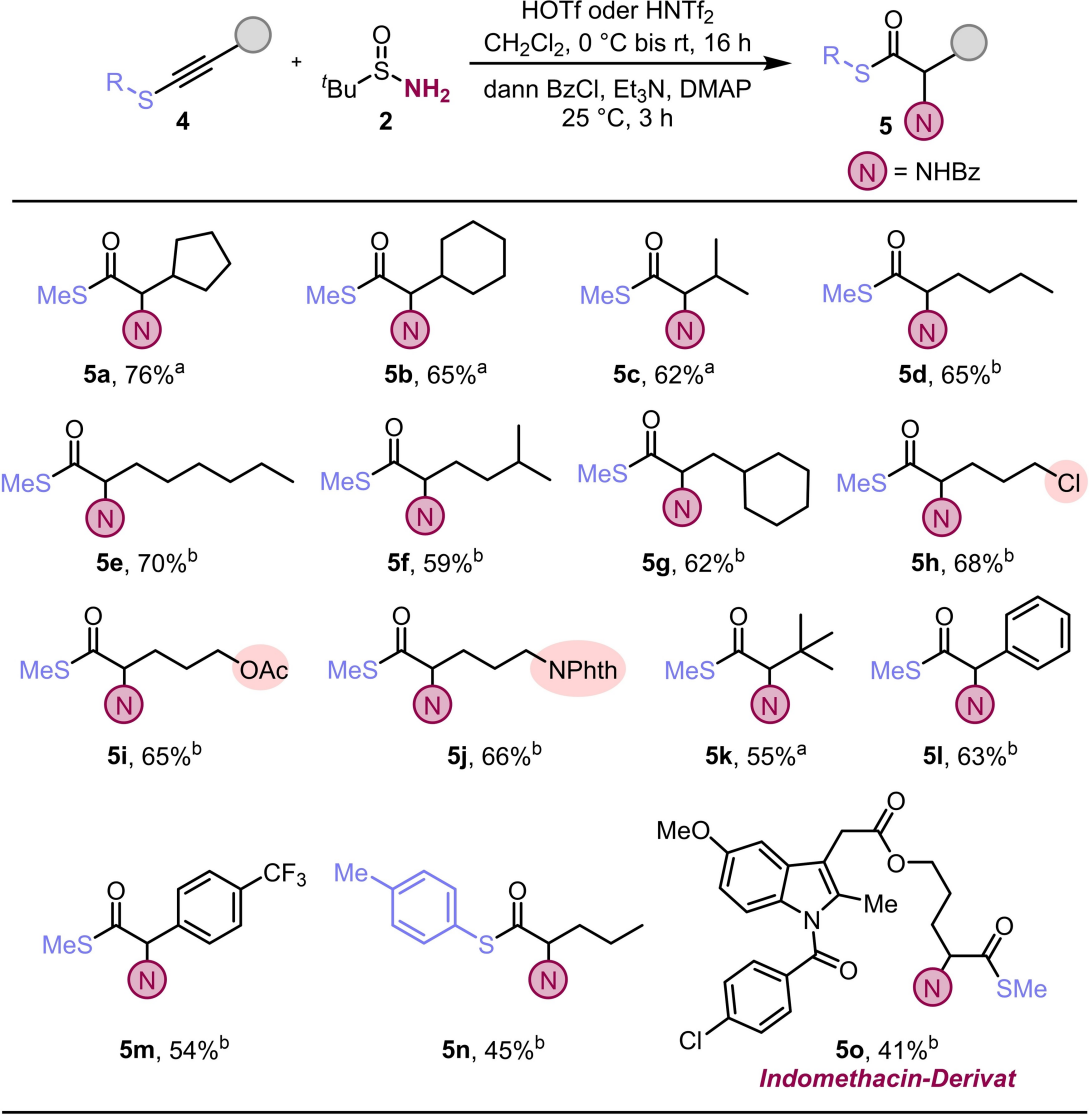
Hydrative Aminierung von Thioalkinen: a) **4** (0.2 mmol,1.0 Äquiv. in 1.0 mL CH_2_Cl_2_), HOTf bei 0 °C für 15 min (1.2 Äquiv.), dann **2** (2.0 Äquiv. in 1.0 mL CH_2_Cl_2_). Nach 16 h, Et_3_N (3.0 Äquiv.), DMAP (5 mol %) und BzCl (3.0 Äquiv.), 25 °C für 3 h. b) **4** (0.2 mmol,1.0 Äquiv. in 1.0 mL CH_2_Cl_2_), HNTf_2_ bei 0 °C für 15 min (0.6 Äquiv. in 0.4 mL CH_2_Cl_2_), dann **2** (1.2 Äquiv. in 0.4 mL CH_2_Cl_2_). Nach 1 h, HNTf_2_ bei 0 °C für 15 min (0.4 Äquiv. in 0.3 mL CH_2_Cl_2_), dann **2** (0.8 Äquiv. in 0.3 mL CH_2_Cl_2_). Nach 16 h, Et_3_N (3.0 Äquiv.), DMAP (5 mol %) und BzCl (3.0 Äquiv.), 25 °C für 3 h.

Um den Mechanismus der Reaktion besser zu verstehen, zogen wir quantenchemische Berechnungen (B3LYP‐D3BJ/def2‐TZVP,SMD(DCM)//B3LYP‐D3BJ/def2‐SVP,SMD(DCM))^[15]^ hinzu (siehe Hintergrundinformationen). Die berechnete Gibbs‐Energie ist in Abbildung 1 dargestellt. Der erste Schritt, für welchen die Bildung eines Keteniminiumions (siehe **A**) durch Inamidaktivierung vorausgesetzt wird,^[16]^ ist ein nukleophiler Angriff des Sulfinamids, welcher die Bildung entweder einer *Z‐* (Profil in blau) oder einer *E*‐konfigurierten Doppelbindung (Profil in lila) zur Folge haben kann. Da das Sulfinamid zwei nukleophile Positionen besitzt, (Sauerstoff und Stickstoff), wurden nukleophile Angriffe sowohl durch O (Schritte **A**→ ***E*** **‐B** und **A**→ ***Z*** **‐B**, rechts) als auch N (Schritte **A**→ ***E*** **‐D** und **A**→ ***Z*** **‐D**, links) in Betracht gezogen. Die Ergebnisse waren eindeutig und in Einklang mit den Experimenten: jener Angriff des Sauerstoffatoms am Keteniminiumion welcher zum *E*‐konfigurierten Intermediat führt (**A**→ ***E*** **‐B**) hat die niedrigste Übergangszustandsenergie (Δ*G* ^≠^(**A**→ ***E*** **‐B**)=5.5 kcal mol^−1^), während der entsprechende Angriff der zur *Z*‐konfigurierten Spezies führt eine um 3.4 kcal mol^−1^ höhere Aktivierungsenergie hat (Δ*G* ^≠^(**A**→ ***Z*** **‐B**)=8.9 kcal mol^−1^). Beide Schritte sind gegenüber dem N‐Angriff kinetisch bevorzugt (Δ*G* ^≠^(**A**→ ***E*** **‐D**)=10.8 kcal mol^−1^ und Δ*G* ^≠^(**A**→ ***Z*** **‐D**)=15.2 kcal mol^−1^). Dies lässt sich auch an den Strukturen der Übergangszustände ablesen: Die Distanz zwischen dem elektrophilen Kohlenstoff und dem Sauerstoff im Übergangszustand **TS**_***E*** **‐AB**_ ist 2.96 Å, während **TS**_***E*** **‐AD**_ eine Distanz von 2.59 Å zwischen Kohlenstoff und Stickstoff aufweist. Dies zeigt, dass der Sauerstoff‐Angriff weniger Annäherung der beiden Reaktionspartner benötigt und somit leichter von statten geht. Zusätzlich zeigt der N‐Angriff eine S−N Bindungsdehnung von über 0.3 Å zwischen **A** (*D*_S−N_=1.70 Å) und den Intermediaten ***E*** **‐D** und ***Z*** **‐D** (*D*_S−N_=2.01 Å und 2.07 Å in ***E*** **‐D** und ***Z*** **‐D**). Im Vergleich dazu ist dieser destabilisierende Effekt im Fall der S−O Bindung und der Bildung der Intermediate ***E*** **‐B** und ***Z*** **‐B** weniger stark ausgeprägt (Dehnung von *D*_S−O_ um 0.21 Å von **A** zu ***E*** **‐B** oder ***Z*** **‐B**). Daraus ergibt sich, dass die Bildung der Intermediate ***E*** **‐B** und ***Z*** **‐B** (durch O‐Angriff) thermodynamisch bevorzugt ist (Δ*G*(**A**→ ***E*** **‐D**)=−17.7 kcal mol^−1^ und Δ*G*(**A**→ ***Z*** **‐D**)=−16.2 kcal mol^−1^, während Δ*G*(**A**→**E‐B**)=−21.5 kcal mol^−1^ und Δ*G*(**A**→ ***Z*** **‐B**)=−21.7 kcal mol^−1^), was die beobachtete Selektivität der Reaktion erklärt. Die beiden Zwischenprodukte haben fast idente thermodynamische Stabilität und können beide eine [2,3]‐sigmatrope Umlagerung eingehen (Schritte ***E*** **‐B**→**C** und ***Z*** **‐B**→**C**), welche in einem einzigen Schritt abläuft. Dieser Schritt führt zum Bruch der S−O Bindung, sowie zur Bildung einer C−N Bindung und somit zu Intermediat **C**. Die Umlagerung hat eine geringe Aktivierungsenergie (Δ*G* ^≠^(***E*** **‐B**→**C**)=9.4 kcal mol^−1^ and Δ*G* ^≠^(***Z*** **‐B**→**C**)=12.7 kcal mol^−1^) und ist ausgesprochen exergon. Intermediat **C** kann dann in Gegenwart von Triethylamin durch Abspaltung des Schwefels zu einem ersten Produkt weiterreagieren (siehe Hintergrundinformationen), wonach die Benzoylierung des Amins das isolierbare Produkt liefert.

**Figure 1:**
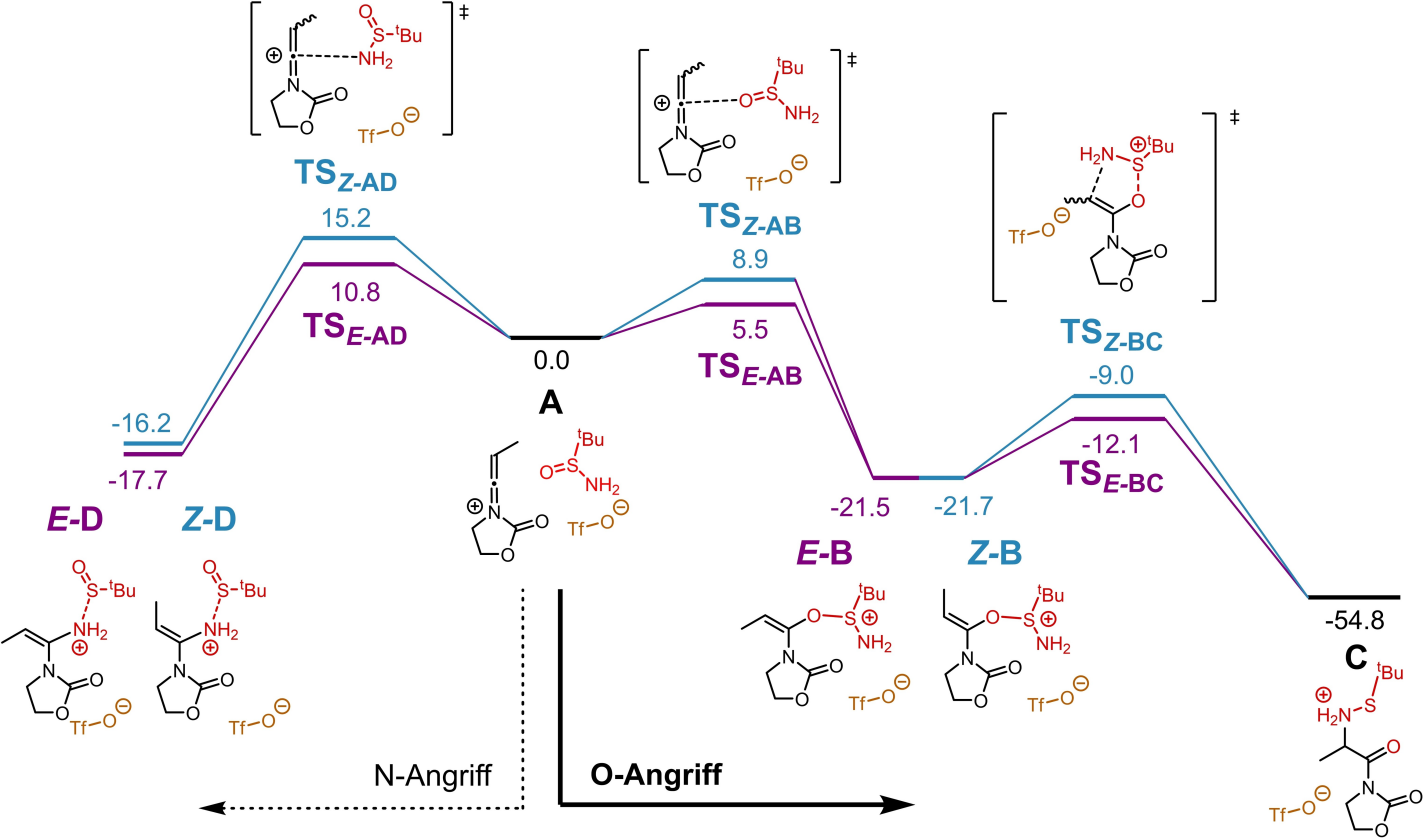
Berechnete Gibbs‐Energie Profile der Reaktion des Sulfinamids mit einem Keteniminiumion: Bildung einer Doppelbindung mit *Z*‐ (Profil in blau) oder *E*‐ (Profil in lila) Konfiguration. Relative Gibbs‐Energien sind in kcal mol^−1^ (298 K) dargestellt. Der Reaktandenkomplex **A** dient als Referenz (0.0 kcal mol^−1^).

Der Pfad der zu den Intermediaten mit *E‐*Konfiguration (Profil in lila) führt zeigt für alle berechneten Reaktionswege niedrigere Aktivierungsenergien als jener der zu *Z‐*Olefinen führt. Dies rührt hauptsächlich von der sterischen Abstoßung zwischen der Methylgruppe des Keteniminiumions und dem Suflinamid her, welches eine *t*Bu‐Gruppe enthält. Die [2,3]‐sigmatrope Umlagerung der Struktur ***E*** **‐B** wurde auch mit alternativen Routen, wie zum Beispiel S−O Bindungsbrüchen und Protonentransfers, verglichen, welche alle weniger wahrscheinlich sind als der hier dargestellte Pfad (siehe Hintergrundinformationen/Abbildung S1).

Enantiomerenreines *tert*‐Butylsulfinamid ist ein bekanntes Auxiliar in der asymmetrischen Synthese von Aminen.^[17]^ Wir untersuchten deshalb die Möglichkeit eines Chiralitätstransfers in unserer [2,3]‐sigmatropen Umlagerung. Ausgezeichnete Enantioselektivität wurde beobachtet wenn sterisch anspruchsvolle Inamide und Thioalkine verwendet wurden (Schema 4A, **(*S*)‐3 h** und **(*S*)‐5 k**); die Selektivität nahm jedoch mit sinkendem sterischen Anspruch rapide ab, vor allem bei Inamiden (**(*S*)‐3 g** und **(*S*)‐3 a**). Die absolute Konfiguration des Produkts **(*S*)‐5 k** wurde durch Röntgenkristallographie bestätigt (CCDC 2208631).^[21]^ Interessanter Weise zeigten Thioalkine durchwegs höhere Enantioselektivität als Inamide, eine Beobachtung die unser Verständnis des Mechanismus der Reaktion beeinflusst. Wenn man den Pfad betrachtet, welcher Intermediate mit einer *E‐*konfigurierten C−C Doppelbindung zeigt (Abbildung 1), ist jener Schritt der die Enantioselektivität bestimmt die [2,3]‐sigmatrope Umlagerung. Um die beobachteten Unterschiede zwischen Inamiden und Thioalkinen zu verstehen, berechneten wir die Gibbs‐Energieunterschiede zwischen den Übergangszuständen die zu den (*S*)*‐* oder (*R*)*‐*Produkten führen (Schema 4B). Für Inamide (Schema 4B, links), zeigt nur der (*R*)*‐*konfigurierte Übergangszustand **TS**_***E‐*** **BC‐(*R*)**_ signifikante sterische Abstoßung zwischen dem Oxazolidinon und der *t*Bu‐Gruppe (orange hervorgehoben). Sowohl der (*S*)*‐* wie auch der (*R*)*‐*Übergangszustand profitieren jedoch von intramolekularen Wasserstoffbrücken (gelb hervorgehoben) zum Carbonyl des Oxazolidinons, von welchen jene im (*R*)‐Übergangszustand etwas stärker ist (0.12 Å kürzer), woraus sich gesamt eine geringe Differenz der Aktivierungsenergien ergibt (ΔΔ*G* ^≠^=1.2 kcal mol^−1^). Diese Wasserstoffbrücke ist im Fall der Thioalkine nicht vorhanden (Schema 4B, rechts), wodurch einzig die sterische Abstoßung zwischen den SMe‐ und *t*Bu‐Gruppen (orange hervorgehoben) im Übergangszustand der zum (*R*)‐Produkt führt (**TS**_***E‐*** **S*M*e‐(*R*)**_) für die Enantioselektivität verantwortlich ist. Dieser Effekt resultiert in einer etwas höheren Differenz der Aktivierungsenergien (ΔΔ*G* ^≠^=1.8 kcal mol^−1^), was direkt mit der experimentell beobachteten, besseren Enantioselektivität korreliert.

**Scheme 4:**
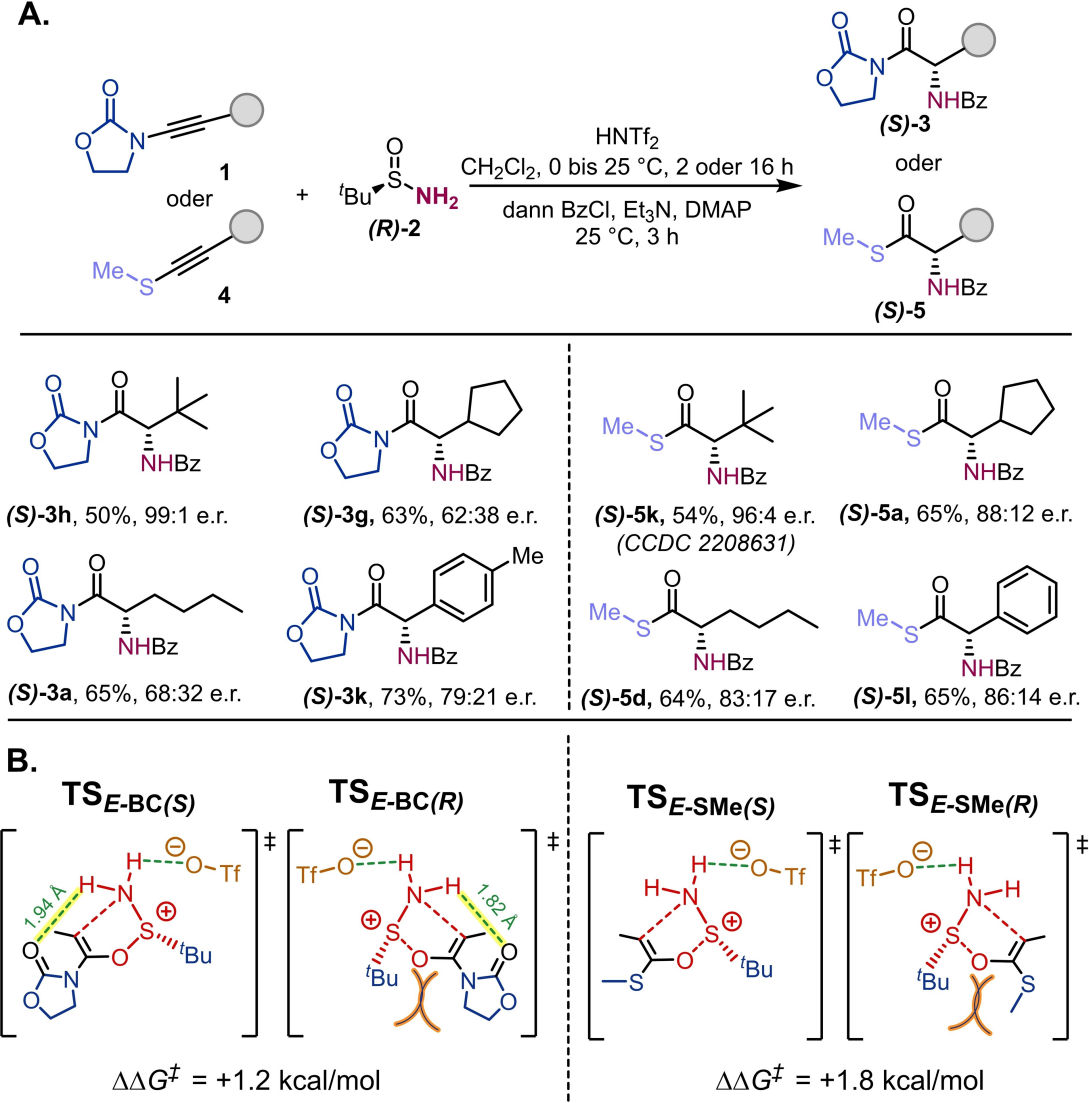
Chiralitätstransfer: A) Reaktion mit enantiomerenreinem (*R*)‐*tert*‐Butylsulfinamid: **1** (0.2 mmol,1.0 Äquiv. in 1.0 mL CH_2_Cl_2_), HNTf_2_ (68.0 mg, 0.24 mmol, 1.2 Äquiv. in 1.0 mL CH_2_Cl_2_) bei 0 °C für 15 min, dann (*R*)‐**2** (48.5 mg, 0.40 mmol, 2.0 Äquiv.). Nach 2 h (für Inamide) oder 16 h (für Thioalkine), Et_3_N (3.0 Äquiv.), DMAP (5 mol %) und BzCl (3.0 Äquiv.), 25 °C für 3 h. Enantiomerenverhältnisse (e.r.) wurden durch chirale HPLC bestimmt. B) Berechnete Gibbs‐Energieunterschiede zwischen den Übergangszuständen die zu (*R*)‐ oder (*S*)‐Produkten führen.

Wir verschrieben uns weiters der Funktionalisierung der gebildeten Produkte (Schema 5), und erkannten, dass das Acyloxazolidinon in Strukturen wie **3 d** sowohl Hydrolyse, als auch Reduktion ermöglicht. Aus diesen Reaktionen gingen einerseits die α‐Aminosäure **6** und andererseits der β‐Aminoalkohol **7** hervor. Im Gegensatz dazu konnte Thioester **5 n** in den α‐Aminoester **8** ^[18]^ und das α‐Aminoamid **9** ^[19]^ überführt werden. Abschließend gelang uns auch eine direkte Kreuzkupplung mit der Boronsäure **11**, um das α‐Aminoketon **10** in ausgezeichneter Ausbeute zu erhalten.^[20]^

**Scheme 5:**
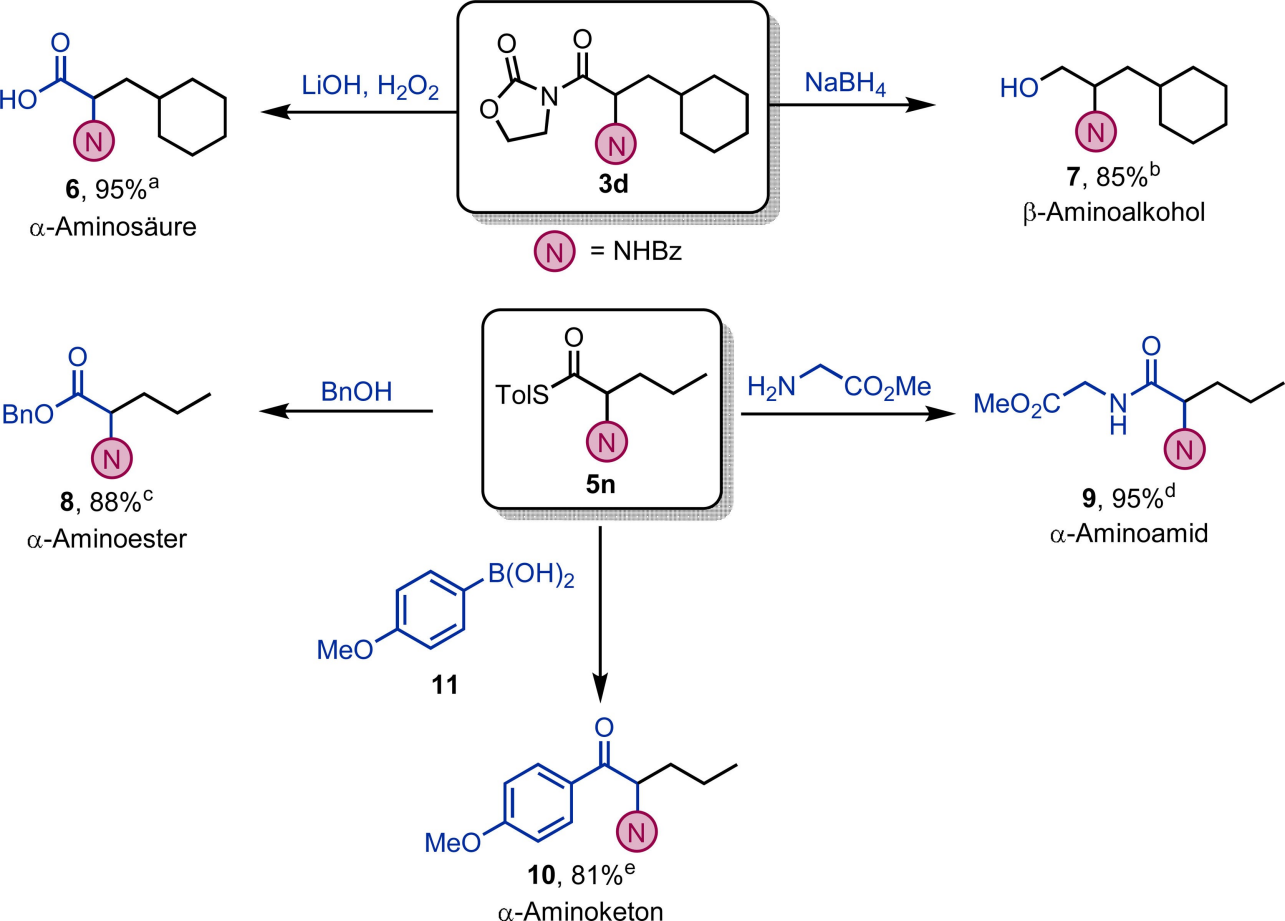
Funktionalisierung: a) **3 d** (1.0 Äquiv.), LiOH (1.1 Äquiv.) und wässr. H_2_O_2_ 30 % (5.0 Äquiv.), THF/H_2_O 3 : 1, 25 °C für 2 h. b) **3 d** (1.0 Äquiv.), NaBH_4_ (5.0 Äquiv.), MeOH, 0 bis 25 °C für 4 h. c) **5 n** (1.0 Äquiv.), BnOH (1.2 Äquiv.), THF, 25 °C für 3 h. d) **5 n** (0.1 mmol), Bis(trimethylsilyl)acetamid (1.5 Äquiv.), Glycin Methylester Hydrochlorid (3.0 Äquiv.), Et_3_N (3.0 Äquiv.), THF, 40 °C für 14 h. e) **5 n** (1.0 Äquiv.), Boronsäure **11** (0.15 mmol), Pd(dba)_2_ (10 mol %), P(OEt)_3_ (20 mol %), CuTC (1.5 Äquiv.), THF, 30 °C für 14 h.

Wir haben hierin eine Methode zur hydrativen Aminierung von Inamiden und Thioalkinen unter milden und metallfreien Bedingungen vorgestellt. Diese praktische Strategie eröffnet einen neuen und bequemen Weg zur Synthese von α‐Aminosäurederivaten von leicht erhältlichen Sulfinamiden, welche als Stickstoffquelle dienen. Computergestützte Berechnungen bestätigen einen Mechanismus welcher auf einer [2,3]‐sigmatropen Sulfoniumumlagerung beruht. Solche Prozesse sind kaum untersucht, was das immense Potential dieser Chemie unterstreicht.

## Interessenkonflikt

Die Autoren erklären, dass keine Interessenkonflikte vorliegen.

## Supporting information

As a service to our authors and readers, this journal provides supporting information supplied by the authors. Such materials are peer reviewed and may be re‐organized for online delivery, but are not copy‐edited or typeset. Technical support issues arising from supporting information (other than missing files) should be addressed to the authors.

Supporting Information

Supporting Information

Supporting Information

Supporting Information

Supporting Information

## Data Availability

Die Daten, die die Ergebnisse dieser Studie unterstützen, sind auf begründete Anfrage beim Autor erhältlich.
